# Practical Application of 3D Printing for Pharmaceuticals in Hospitals and Pharmacies

**DOI:** 10.3390/pharmaceutics15071877

**Published:** 2023-07-04

**Authors:** Kampanart Huanbutta, Kanokporn Burapapadh, Pornsak Sriamornsak, Tanikan Sangnim

**Affiliations:** 1 Department of Manufacturing Pharmacy, College of Pharmacy, Rangsit University, Pathum Thani 12000, Thailand; 2 Department of Industrial Pharmacy, Faculty of Pharmacy, Silpakorn University, Nakhon Pathom 73000, Thailand; 3 Academy of Science, The Royal Society of Thailand, Bangkok 10300, Thailand; 4 Faculty of Pharmaceutical Sciences, Burapha University, 169, Saensook, Muang, Chonburi 20131, Thailand

**Keywords:** 3D printing, additive manufacturing, personalized medicine, telepharmacy

## Abstract

Three-dimensional (3D) printing is an unrivaled technique that uses computer-aided design and programming to create 3D products by stacking materials on a substrate. Today, 3D printing technology is used in the whole drug development process, from preclinical research to clinical trials to frontline medical treatment. From 2009 to 2020, the number of research articles on 3D printing in healthcare applications surged from around 10 to 2000. Three-dimensional printing technology has been applied to several kinds of drug delivery systems, such as oral controlled release systems, micropills, microchips, implants, microneedles, rapid dissolving tablets, and multiphase release dosage forms. Compared with conventional manufacturing methods of pharmaceutical products, 3D printing has many advantages, including high production rates due to the flexible operating systems and high drug loading with the desired precision and accuracy for potent drugs administered in small doses. The cost of production via 3D printing can be decreased by reducing material wastage, and the process can be adapted to multiple classes of pharmaceutically active ingredients, including those with poor solubility. Although several studies have addressed the benefits of 3D printing technology, hospitals and pharmacies have only implemented this process for a small number of practical applications. This article discusses recent 3D printing applications in hospitals and pharmacies for medicinal preparation. The article also covers the potential future applications of 3D printing in pharmaceuticals.

## 1. Introduction

Three-dimensional (3D) printing, also called additive manufacturing, produces 3D objects from digital models by fusing, depositing, or solidifying various types of materials layer by layer [1]. For most 3D printing procedures, CAD (computer-aided design) software is used to create the design, which is then sent to the appropriate 3D printing (or slicing) program using a standard tessellation language (.stl) file. Using slicing software, a 3D design is divided into layers of appropriate thickness for the specific 3D printer. After the design is divided into parts, the 3D printer constructs the design to the desired size and form. This technology was invented more than 50 years ago [1] and has been applied in several fields, including aerospace [2], automotive [3], food [4], healthcare [5], architecture [6], construction [7], and electronic industries [8]. Three-dimensional printing is attractive for several reasons. First, the cost of a 3D printer is affordable, making it accessible to a large number of end users. Second, several printing techniques can be used for a variety of applications and printing materials. The manufacturing period and printing quality are now superior to those of the past [9]. Therefore, it is unsurprising that 3D printing has begun to pervade academic research, with a significant influence in fields that can swiftly prototype and develop products [10]. 

Three-dimensional printers have been utilized in a variety of ways for pharmaceutical and medical applications, including the fabrication of tissues and organs [11], the creation of customized prosthetics [12], the use of patient-specific implants, and the preparation of complex or personalized drug delivery systems [10,13,14,15]. In addition, this technology can be applied to the concepts of telepharmacy and telemedicine to prescribe medications over long distances [16]. Recent scholarly publications and patents pertaining to the healthcare application of 3D printing technology have surpassed 6400 research articles and 31 patents globally in 2020 [17]. Moreover, 3D printing is increasingly applied in drug stores and hospitals. This is because 3D printers are portable, small, and easy to use, which makes them ideal for usage in hospital wards, in-patient pharmacies, specialist clinics, and community pharmacies. Additionally, they can prepare medications on demand [18].

This article provides an overview of the fundamental theory behind 3D printing, the main 3D printing methods that are used to produce pharmaceuticals, potential applications of this technology in healthcare settings such as hospitals and pharmacies, the current state of 3D printing pertaining to pharmaceutical applications and issues, and challenges that are associated with this technology, as well as its future potential. 

## 2. Type and Printing Technique of 3D Printers Used in Pharmaceutical Applications

**Figure 1:**
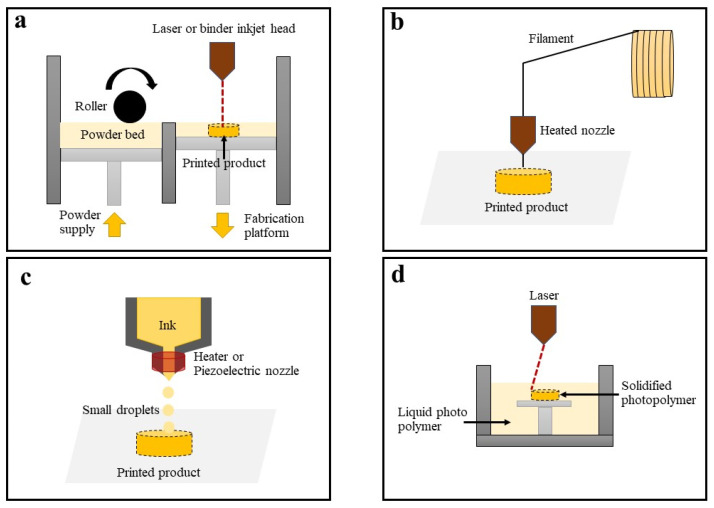
Schematic working process of 3D printing techniques, including (**a**) powder-based 3D printing, (**b**) extrusion-based 3D printer, (**c**) inkjet-based 3D printing, and (**d**) laser-based 3D printing.

Several types of 3D printing techniques have been utilized in the pharmaceutical industry, with the technique chosen depending on the specific application and type of the manufactured product (Figure 1). In the pharmaceutical industry, only a few of the numerous 3D printing techniques have been implemented. The technique employed can vary depending on the specific requirements of the manufactured product and the materials employed. The following examples of 3D printing techniques have been implemented.

### 2.1. Powder-Based 3D Printing

A powder-based 3D-printing technique whereby a binder solution or laser is blasted/beamed onto a powder bed to bind the powder together and create a 3D-printed structure [19,20]. Typically, this system includes a binder solution reservoir to store the binder/ink, a powder reservoir, and a build platform for the printing process, as illustrated in Figure 1a [21]. Various pharmaceutical products, including fast-dissolving tablets [22], complex medications with zero-order release [23], and novel-shaped tablets [21], have been created using powder-based 3D printing.

### 2.2. Extrusion-Based 3D Printer

Extrusion-based 3D printing uses a heated nozzle to extrude a continuous strand of molten polymer, which is then layered and cooled to form a 3D object (Figure 1b) [15]. This technique is divided into two methods: semi-solid extrusion and fused deposition modeling [24]. The technique is uncomplicated, and controlling the printing parameters is simple. Therefore, extrusion-based 3D printing has been extensively utilized in pharmaceutical research. Various pharmaceutical products, such as immediate-release tablets [25], floating tablets [1,14], colonic drug delivery [26], and modified-release tablets [13], have been prepared using this technique. However, concerns exist about the use of this technique. The active ingredient should be stable at high temperatures because of the use of a heated nozzle for printing, and the medications must be prepped with filaments before printing.

### 2.3. Inkjet-Based 3D Printing or Digital Inkjet Printing

Continuous inkjet printing and drop-on-demand printing are two types of technologies included in inkjet-based 3D printing systems. While continuous inkjet printing technology uses a high-pressure pump to produce an uninterrupted flow of ink through an orifice ranging from 50 to 80 microns in diameter, drop-on-demand printing technology creates droplets ranging from 10 to 50 microns in size with volumes ranging from 1 to 70 picoliters [27]. This technique involves using an inkjet printhead to deposit layers of a photopolymer resin, which is then cured using a UV light source to form a 3D object, as shown in Figure 1c. This printing technique is suitable for labile drugs or heat-sensitive materials [28]. Various pharmaceutical dosage forms have been prepared using this technique, including tablets [28], implants [29], and orodispersible films [30].

### 2.4. Laser-Based 3D Printing

This technique is also known as selective laser sintering (SLS), laser beam melting, or stereolithography (SLA). The fabrication of a 3D object by SLA is based on the controlled solidification of a liquid resin via photopolymerization [31]. This method provides a highly accurate and precise scale of printing. Consequently, it has been used to manufacture pharmaceutical products that require a high level of precision, such as microneedles [32]. 

## 3. Printing Materials Used in Pharmaceutical 3D Printing

Printing materials can signify the release profiles, physical properties, stability, printer setting parameters, and printing detail. Furthermore, different printing techniques require printing materials with distinct properties. The following is an example of 3D-printed materials used in oral pharmaceutical formulations.

### 3.1. Lactose

Lactose can be used as a filler or diluent in 3D powder bed printing, or SLA, for pharmaceuticals. It can enhance the compactability, physical stability, and drug solubility of the powder mixture used in 3D-printed tablets [33,34]. Generally, a lactose monohydrate binder blend is preferred for 3D powder bed printing due to the fact that a proper binder can enhance the mechanical properties of the tablet. A hydrophilic and hydrophobic API was successfully formulated into a printed tablet using lactose as a diluent due to its high solubility.

### 3.2. Polylactic Acid (PLA) 

PLA is a biodegradable polymer that is often used in the 3D printing of implants, scaffolds, and drug delivery systems. It is accepted by the United States Food and Drug Administration (FDA) and is considered safe for use [35]. PLA has sufficient thermoplastic properties to be printed using the fused deposition modeling (FDM) technique [36,37]. The proper grade and molecular weight of PLA, together with a proper plasticizer, can be prepared for the filaments. PLA can be formulated with various drugs and excipients to achieve the desired release kinetics. However, PLA has a slower rate of degradation, making it suitable for sustained drug release [13,38].

### 3.3. Polyvinyl Alcohol (PVA) 

PVA is a water-soluble thermoplastic polymer that is often used as a filler or binder in 3D printing tablets due to its biodegradability, biocompatibility, and dissolvability. It can improve the mechanical strength and drug release properties of the printed dosage forms. PVA can be printed using extrusion-based [14] or inkjet-based techniques [39]. For example, Goyanes and his team manufactured personalized oral caplets via fused deposition modeling using PVA filaments loaded with paracetamol or caffeine [40]. Kampanart and his colleagues also used PVA to produce tablet housing in order to control the drug’s release profile and allow for zero-order release, as well as to lift the core tablet on gastric fluid for at least eight hours [14]. In addition to oral dosage forms, 3D-printed PVA is applicable to suppository dosage forms. Tatsuaki and his colleagues have developed a water-soluble polymer (polyvinyl alcohol) suppository shell for controlled drug release [41].

### 3.4. Hydroxypropyl Methylcellulose (HPMC) 

HPMC is a cellulose-based polymer that is commonly used in 3D-printed dosage forms as a binder, diluent, supporting structure, and sustained-release agent. In comparison to other 3D printing materials, HMPC is distinguished by its dissolvability and low toxicity. Generally, HPMC can be printed in three dimensions using an extrusion technique [42]. Prashant and co-workers applied HPMC and methyl cellulose as biodegradable support structures. It was discovered that HPMC can be printed while maintaining the hollow structure of the primary printed product, and it can be easily removed from the building material by dissolving it in water [42]. Yiliang and his team used an extrusion-based 3D printer to prepare semi-solid tablets with different drug-loading dosages at ambient temperature. The active pharmaceutical ingredient, theophylline, was incorporated into hydrogels prepared with HPMC K4M or E4M for a prolonged drug release profile [43]. 

### 3.5. Gelatin

Gelatin is a natural protein-based biopolymer derived from collagen, which is typically derived from bovine or porcine skin and bones. Due to its biocompatibility, biodegradability, and simplicity of processing, it has been used for decades in a number of pharmaceutical formulations. Gelatin can be used as a bio-ink [44] or a printable material in 3D printing to construct complex structures and drug delivery systems [45]. Due to the gelatin’s pliability, elasticity, and hydrophilicity, it has been utilized as a 3D-printed biodegradable excipient in gummy antiepileptic drug formulations for pediatric patients via the extrusion base technique [45]. 

### 3.6. Polyethylene Glycol (PEG)

PEG is a versatile polymer that can be used in 3D printing in pharmaceutical applications. It is a water-soluble, biocompatible, and biodegradable material that offers several advantages for drug delivery and tissue engineering [46]. Hsin-Yun Hsu used the drop printing technique to prepare naproxen/PEG 3350 solid dispersions with PEG coatings of different molecular weights, resulting in precise dosages and predictable compositional uniformity of API in three-dimensional structures [47].

## 4. Journey of 3D Printing in Pharmaceutical Application

The pharmaceutical industry has used 3D printing technology for several decades, but it has only recently begun to gain widespread adoption. One of the early pioneers of 3D printing was Charles (Chuck) Hull, who filed a patent application in 1986 for the use of UV lamps to cure photosensitive resin layer by layer to create small custom parts [48]. Therefore, the first 3D printer was based on stereolithography, after which a selective laser sintering technique was developed. The field of 3D printing did not become popular until the last two decades. In 2000, the first research article on oral dosage forms fabricated by 3D printing was published in the *Journal of Controlled Release.* In this study, Katstra and her team applied powder-based 3D printing to produce oral tablet dosage forms. Various types of binders, such as Eudragit E100, were added in different amounts to the PVP powder bed layer. Chlorpheniramine maleate was selected as the model drug, and the drug release profile was assessed to reveal the effect of binder type and amount on drug release behavior [49]. After that, in 2009, a biotechnology company called Organovo developed the first commercially available 3D bioprinter that could produce functional human tissues and organs [50]. This achievement represented a significant step forward in the evolution of 3D printing in the biopharmaceutical industry. 

In 2015, the Food and Drug Administration (FDA) granted approval for the first 3D-printed drug, which was a seizure medication called Spritam^®^ (levetiracetam) [51]. Spritam^®^ is available in orodispersible tablet (ODT) dosage form. It was produced using the powder bed technique of 3D printing by adding liquid binder layer by layer. Spritam disintegrates extremely rapidly in comparison to other ODTs due to the absence of compressive force during tablet preparation. This was a significant milestone in the approval process of 3D-printed pharmaceutical products. The FDA also approved the first-ever 3D-printed medical device, which was a tracheal splint intended to treat an extremely uncommon respiratory condition in newborns [52,53]. In 2016, the world’s first 3D-printed cranial implant, a titanium cranial implant used to repair skull defects, was approved by the FDA [54]. In 2017, the FDA initiated a pilot program to investigate the viability of employing 3D printing in the production of medical goods [55]. In 2018, the FDA approved the first drug-eluting stent produced using 3D printing technology [56]. Recently, the first once-a-day single inhaler triple therapy, a device used to deliver medication to the lungs, for treating both asthma and chronic obstructive pulmonary disease (COPD), received approval from the FDA in 2020 [57]. 

**Figure 2:**
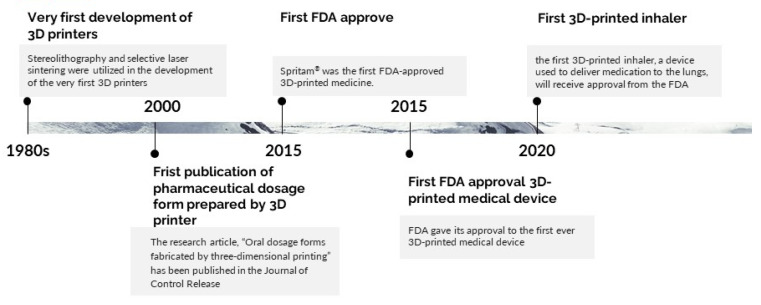
Timeline highlighting several significant turning points in the evolution of 3D printing in the medical and pharmaceutical industry.

A significant amount of research and development has since been conducted in this area, and many pharmaceutical companies have started to explore the use of 3D printing for a variety of applications, including drug dosage forms, drug delivery devices, and medical devices and implants [58]. The following is a timeline highlighting several significant turning points in the evolution of 3D printing in the pharmaceutical industry (Figure 2). Thus, this technology will continue to evolve and be used in new and innovative ways in the coming years. 

## 5. Advantages and Limitations of 3D Printing in Pharmaceutical Application

The use of 3D printing in the pharmaceutical industry is accelerating as a result of the numerous benefits that it offers. However, several constraints are associated with the use of 3D printing in the production of pharmaceuticals. The advantages and disadvantages of 3D printing in pharmaceutical applications are outlined in Table 1.

## 6. Pharmaceutical Application of 3D Printing in Hospitals and Pharmacies

In hospitals and pharmacies, 3D printing has the potential to significantly advance drug development, personalized medicine, medical devices, and drug delivery. By harnessing the power of additive manufacturing, researchers and pharmaceutical companies can create patient-specific drug formulations and dosage forms, thereby enhancing drug solubility, controlled release profiles, advanced medical devices, and the possibility of solidifying the concept of telepharmacy. In addition, 3D printing enables the production of personalized medications based on patient-specific needs, enabling the development of customized dosage forms and streamlined medication regimens. In the domain of medical devices and implants, 3D printing has revolutionized production by producing anatomically precise models for surgical planning and patient-specific implants that improve comfort and quality of life. By fabricating sophisticated drug delivery systems, such as microneedle patches and inhalable formulations, it is possible to exert precise control over drug release rates, dosages, and spatial distribution. Additionally, 3D printing facilitates rapid prototyping for clinical trials and research, allowing for the evaluation and optimization of novel formulations and the creation of tissue models that closely resemble human physiology, thereby reducing the need for animal testing. Moreover, 3D printing automates compounding processes in pharmacies, ensuring precise dosages and drug combinations and enhancing precision, efficiency, and patient safety. While regulatory considerations, material selection, and quality control remain obstacles, 3D printing is a promising technology for revolutionizing pharmaceutical innovation, due to ongoing advancements [66]. Examples of real-world 3D printing applications in hospitals and pharmacies are provided in Table 2.

### 6.1. Personalized Medicine

A major benefit of 3D-printing medicines is the potential to adjust the individual dose. Therefore, this technology is advantageous for treating patients with complex diseases who need personalized medication. Warfarin is one drug where the dose needs to be adjusted due to its narrow therapeutic index [80]. Dose titration of the drug is achieved by monitoring the international normalized ratio. Even though the strength of a commercial warfarin tablet varies from 2 to 10 mg, tablet splitting is still common a practice in hospital wards to adjust the dose for each patient [81]. The use of 3D printing to tailor the warfarin dose was reported for oral films and tablets [82,83,84,85]. Orally disintegrating tablets of warfarin were prepared using binder jet printing [84]. The tablets had an accurate drug content, acceptable mechanical strength, and a rapid disintegration time. As powder bed fusion was used to develop the first commercial 3D printing tablet, the application of this in the pharmaceutical industry is indisputable. However, the administration of powder bed fusion technology in healthcare units is not easily accessible. The expensive processing tools and difficulty in relocating the processing unit have become crucial obstacles to using this technology in hospitals and pharmacies. 

Other technologies that have been reported for the dosing adjustment of warfarin rely on extrusion-based systems. Fused deposition modeling (FDM), which is simpler and more cost-effective [86], was used to prepare various doses of warfarin tablets [85]. The 3D-printed tablets had a range of dose accuracy between 91.5% and 102.4%, with a high correlation between the target and achieved dose, even at very low doses of 500 µg. Due to the drug-loading method, warfarin dispersed in the methacrylic polymer matrix is in an amorphous form. Thus, the pharmacokinetic behavior of warfarin in the animal model was altered for sustained drug release to enable a lower C_max_ and longer T_max_ in comparison with that of the warfarin solution.

Orodispersible films containing various doses of warfarin intended for use in pediatric and geriatric patients were successfully developed using semi-solid extrusion printing (SSE) [82,83]. Warfarin was dispersed in a matrix of hydroxypropyl cellulose, and the dose was controlled by an adjustment of the film size. The accuracy of the dose was found to be superior in comparison with that of unit dose sachets prepared by manual compounding [82]. Furthermore, a QR code containing dosage information can be incorporated into the 3D-printed films to avoid medication errors.

The first clinical study on the application of a 3D-printed dosage form in a hospital was reported in 2019 [67]. Chewable 3D-printed tablets, so-called printlets, prepared by SSE 3D printing, were used to treat pediatric patients (aged 3–16 years) with maple syrup urine disease, where supplementation of valine and isoleucine must be strictly controlled throughout life [87]. The isoleucine-loaded printlets were produced in the hospital with various doses and flavors. The drug content, in vitro dissolution, and stability of the printlets were investigated to ensure the efficacy of the dosage form. Isoleucine blood levels were evaluated using the dried blood spot method, and the acceptability of the 3D-printed formulation was evaluated in comparison with that of isoleucine capsules prepared by manual compounding. Isoleucine blood levels from both capsules and printlets were within the target range. However, the printlets were found to be more appropriate and acceptable for pediatric patients due to their candy-like characteristics. Based on this study, dose adjustments of the drug could be promptly manipulated for specific patients. Thus, the possibility of using 3D printing technology for dose adjustment was clearly demonstrated.

Another study that applied 3D printing in a hospital setting was reported in 2020 [72]. Commercial spinorolactone tablets were ground and blended with other excipients before being printed into subdivided tablets using an extrusion-based 3D printer. The printer was located in a hospital pharmacy within an ISO5-level environment and was operated by well-trained operators. The 3D-printed tablets were qualified by a pharmacist before being dispensed predissolved in water to pediatric inpatients. This study reported positive responses from both patients and healthcare staff. However, the acceptance in comparison with commercial tablets and the pharmacokinetic behaviors of the drug after administration were not mentioned.

### 6.2. Multiple Medications

**Figure 3:**
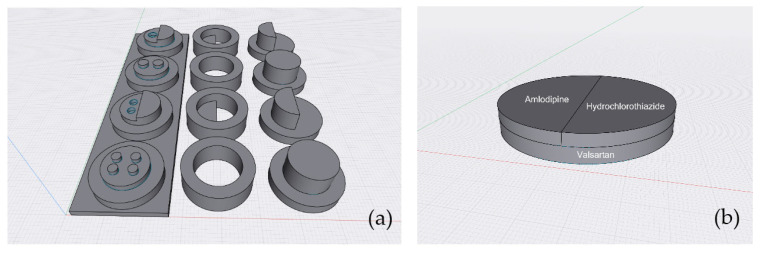
(**a**) Mold design and (**b**) assembled tablet of the polypill.

Multiple medications or polypharmacy are commonly defined as the regular use of at least five medications in an individual patient [88]. This situation is frequently found in elderly or young patients with specific conditions. In addition to causing confusion and noncompliance problems, patients also face the risk of adverse medical outcomes. To solve the polypharmacy problem, several studies have combined various active pharmaceutical ingredients into one tablet [89,90]. Multiple drugs, dosages, and drug-release profiles can be combined into one dosage unit of a “polypill” fabricated using 3D printing technology [90,91]. Sangnim and her colleagues developed an assembled polypill prepared from a 3D-printed mold inspired by Lego bricks (Figure 3a). This polypill was composed of three commonly used hypertension drugs (amlodipine, hydrochlorothiazide, and valsartan), as illustrated in Figure 3b. This innovation could potentially increase patient compliance, particularly among the elderly [92]. Mark and his team explored the perceptions and preferences of polypharmacy patients regarding 3D-printed medicine, including their acceptance of patient-designed medication. The patients were asked about their perceptions and preferences regarding 3D-printed solid dosage forms and were shown various shapes, colors, embossing patterns, and polypills. The findings revealed that the patients’ perspectives and preferences regarding 3D-printed medicine varied. Appealing, swallowing, handling, comprehension, and psychological factors affect a patient’s perspective [68]. However, no clinical studies on the administration of polypills in patients have been conducted to date. Knowledge of and trust in 3D printing technology are suggested as important factors in determining whether geriatric patients accept or reject this technology [68]. Therefore, the advice from pharmacists at the point of care plays an important role in the success or failure of bringing the personalized 3D-printed dosage form to reality.

### 6.3. Drug-Loaded Medical Devices

The advent of 3D printing has changed the development of medical devices, particularly drug delivery systems. Many drug-loaded medical devices, such as implants [93,94,95], inhalers [96,97], transdermal patches [98,99,100], and orthodontic retainers [78], have been made via 3D printing. For implantable drug delivery systems, the use of 3D printing for drug-eluting implants combines the benefits of targeted local drug therapy over longer periods of time at the precise location of the disease with a manufacturing technique that allows easy modification of the implant shape to meet the individual needs of each patient. Until recently, research has concentrated on a variety of features of this topic, such as 3D printing using various materials or printing procedures, to create implants with distinct forms, mechanical properties, and release profiles. For example, Sarah and colleagues developed a biodegradable subcutaneous implant for prolonged drug delivery using 3D printing. Five implant designs were created. It was found that the release rate varied based on the implant design and drug characteristics. In addition, a rate-controlling membrane was constructed, which further delayed the release from the manufactured implants, indicating its potential utility for chronic illnesses [101].

**Figure 4:**
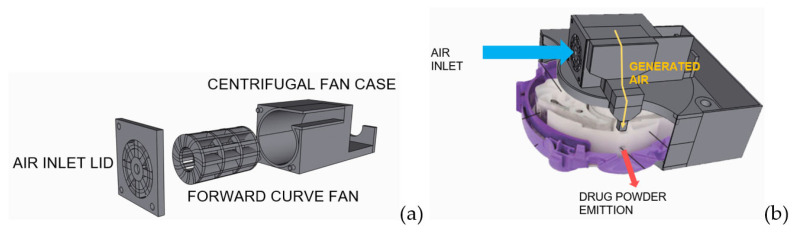
(**a**) Fan design and (**b**) working diagram of the add-on device for dry powder inhalers (Accuhaler).

Apart from the implantable drug delivery system of the dosage form, Suwanpitak and his team developed an add-on device for dry powder inhalers (Accuhaler) prepared via 3D printing. This add-on device could improve drug administration efficiency in patients with limited inspiratory capacity, including young children, the elderly, and those with COPD. The add-on device consisted of a motor and fan that could be attached to the Accuhaler (Figure 4a,b). The use of the add-on device in conjunction with a sufficient inhalation flow rate led to an increase in the DPI-emitted drug dose for patients with low inspiratory rates, thereby facilitating the administration of adequate drug doses for improved treatment outcomes [10].

For advanced transdermal medication delivery applications, 2D and 3D printing is used. The applicability of several printing technologies has been researched for the direct or indirect printing of microneedle arrays or for the modification of their surface through drug-containing coatings [100]. Using a digital light processing 3D printer, Lim and coworkers created a splint with microneedles for the treatment of trigger fingers. Using the device to puncture the skin prior to the topical administration of diclofenac gel, they successfully increased the drug amount that permeated the skin demonstrating that 3D printed patient-specific microneedle devices can be effective in systems designed using the ‘poke-and-patch’ strategy [102]. Personalized orthodontic retainers containing clonidine hydrochloride have been fabricated using a 3D printing process involving hot–melt extrusion. This invention addresses the unanticipated side effects caused by the in vivo burst release of clonidine hydrochloride [78]. Another innovative 3D-printed medical device for drug delivery systems is a customizable design mouthguard. The study showed the first-in-human use of the 3D-printed mouthguard and demonstrated the immense potential of 3D printing as a platform for the development and translation of next-generation drug delivery devices for personalized therapy [77].

### 6.4. Customized Design

One benefit of 3D printing technology is its ability to construct various shapes of dosage units to suit patient anatomy and preferences. A standard FDM printer, inspired by Starmix^®^ gummy sweets structures, was used in the production of taste-masked chewable tablets for pediatric patients [71]. In addition to drug-infused gummy candies, a variety of pediatric pharmaceutical dosage forms have been created and manufactured utilizing various 3D printing processes. Januskaite and colleagues evaluated the preference of children for 3D-printed tablets (Printlets^TM^) made using four distinct 3D printing procedures, namely digital light processing, selective laser sintering, semi-solid extrusion, and fused deposition modeling. The digital light processing printlets were determined to be the most visually appealing to the children. Digital light processing provides an appropriate visual appearance (color and shape) for printlets, which is crucial for a child’s first impression. However, pleasant flavor and chewing texture are two essential aspects that must be considered when developing formulations for children [79]. Another actual case study that used 3D-printed dosage forms in a trial was the study by Goyanes and his colleagues. They used a semi-solid extrusion 3D printer to prepare a small batch of isoleucine in the form of a chewable formulation at the point of dispensing (in a hospital setting) to treat a rare metabolic disease in children [67]. 

In geriatric patients, the most typical criterium is a reasonable dosage form size that is easy to swallow, which is relatively comparable to the requirements for pediatric dosage forms. Therefore, fast-dissolving tablets and films have been invented for elderly patients [30,103,104,105]. Several 3D printing techniques have been utilized to manufacture pharmaceutical dose forms with rapid dissolution or disintegration. Sorato and his team, for instance, employed the 3D printing process of fused deposition modeling to create fast-dissolving oral dose forms. Typically, fused deposition modeling 3D printing technology can only produce solid objects, which might result in cracking, chipping, and difficulty dissolving [15]. This is due to the fact that the printing materials used for fused deposition modeling are primarily thermoplastic polymers with insoluble or slow-dissolving characteristics. Thus, the authors added sugar alcohol to a formulation of poly (vinyl alcohol) filaments, resulting in a faster drug release [106]. In addition to the rapid dissolution formulation property, the concept of drug identification by color or shape connected to pharmacological categories is favored for geriatric patients. The embossing designs, which indicated the time for administration for an individual person, are also suggested to be useful for patients with cognitive impairments and health assistants who have to provide medication to these patients [63]. Moreover, 3D-printed tablets with embossing designs were developed for patients with visual impairment and blindness. Tablets embossed with Braille and Moon patterns were prepared using a desktop SLS printer. In addition to helping patients identify their medications and decreasing the risk of medical errors, patient compliance may be improved [75].

Lastly, 3D printing can be applied to scaffold applications. The 3D printing technique has several advantages over traditional scaffold preparation. For example, 3D printing allows for the fabrication of polymeric cellular materials with empty spaces in a well-defined periodic structure. Moreover, it can create scaffold tissues of precise and individualized size and shape for each individual patient. And recent 3D printing technology demonstrates the possibility of industrial scaffold production [107]. Tammaro and his team produced thermoplastic polymeric (polylactic acid (PLA)) foams with solubilized physical expanding agents using a continuous 3D printing process. In terms of morphology, the resultant foamed strands and hierarchical structures are novel and exhibit controlled local porosity and superior mechanical properties that are suitable for scaffold structures [107].

### 6.5. Telepharmacy

**Figure 5:**
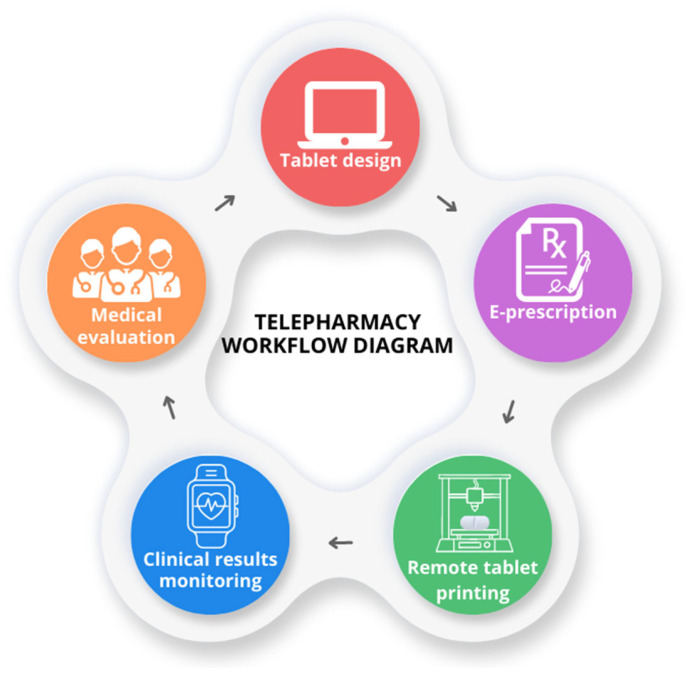
3D printing applications in telepharmacy workflow.

The delivery of pharmacy services at a distance can be accomplished using telecommunication and information technologies, such as videoconferencing and electronic health records. This practice is known as “telepharmacy” [108]. Telepharmacy refers to the remote provision of pharmacy services, often in underserved areas where access to healthcare facilities is limited. Telepharmacy benefits patients by enabling remote pharmacies to overcome logistical challenges and improve patient care. Three-dimensional printing technology may have the potential to be utilized in telepharmacy in various approaches, as shown in Figure 5. Three-dimensional printing can be used to prepare personalized medication for patients who are located at a distance from one another. Adjustments to the dosage of the medication can be made in accordance with the clinical results, such as changes in the patient’s heart rate, blood pressure, and blood sugar level. These clinical outcomes can be remotely monitored by wearable devices, such as sensors or patches [109,110]. Patients with chronic disease conditions that are stable and under their control can greatly benefit from this concept. This can give patients the convenience to reduce their travel, which also helps to limit the number of people in the hospital [111]. In the event of a natural disaster or other emergency that disrupts supply chains, 3D printers can be used to produce essential medications and medical supplies on-site, ensuring continued access to healthcare.

In addition, 3D printing can help telepharmacy facilities provide important medical devices. With the use of 3D printers, customized medical devices and aids, such as pill organizers, inhaler spacers, prosthetic components, and orthopedic supports, can be manufactured. These devices can be adapted to the specific requirements of each patient, thereby enhancing patient comfort and adherence to treatment plans. Telepharmacy facilities can collaborate with remote healthcare professionals using 3D printing technology. Experts can provide guidance, evaluate prescriptions, and guarantee the quality and safety of 3D-printed medications and medical devices by sharing their digital designs. This collaboration improves patient outcomes by facilitating access to specialized knowledge and expertise.

## 7. Practical Considerations for Hospitals and Pharmacies

The pharmaceutical dosage from 3D printing is a relatively new technique that provides numerous potential advantages, including tailored medicine therapy and a quicker time to market for new drugs. But hospitals and pharmacies must also be mindful of a variety of practical problems when employing this technology. The primary concerns are the equipment and materials. Hospitals and pharmacies must invest in 3D printers and in the materials required to create the desired pharmaceutical compositions. The printers must be able to print at a high resolution to ensure dosage accuracy, and the materials must be of high quality and purity to guarantee safety and efficacy. In addition, the availability of sufficient pharmaceutical-grade materials for 3D printing is a formidable obstacle. The utilized materials must be compatible with the medicine, biocompatible, biodegradable, and acceptable for 3D printing production. In addition, they must not generate potentially toxic substances during the printing process [112]. Quality control and safety are also crucial since they verify that the printed medications fit the appropriate requirements and contain the correct amount of medication. Consideration must be given to the drug’s stability over time and its degradation due to heating, pressure, and solvent during the printing process [113]. The training and education of healthcare workers, including pharmacists, physicians, nurses, and technicians, are essential. Hospitals and pharmacies must educate and train their employees on how to use 3D printing technology safely and effectively [59]. Overall, 3D printing of pharmaceutical dosage forms is a promising technology that offers many potential benefits; nevertheless, in order to ensure that its adoption is both safe and successful, careful consideration of the practical factors mentioned above is required.

Numerous 3D-printed pharmaceutical products are currently undergoing clinical trials. The FabRx team conducted a clinical trial in which they utilized personalized 3D-printed dosage forms to treat children with maple syrup urine disease, a rare metabolic disorder. This trial marked the first time such dosage forms were used in a clinical study [67]. Triastek, Inc., a pharmaceutical company based in China, announced that the US Food and Drug Administration (FDA) has approved its investigational new drug (IND) application to initiate clinical studies of T21, a 3D-printed medicine that can target specific segments in the colon to deliver oral ulcerative colitis drugs more safely [114]. In addition to facilitating clinical trials of pharmaceutical products, expanding the use and research of 3D printing requires clinical toxicity studies of 3D printing materials [115]. These examples illustrate the accelerating adoption of 3D printing in point-of-care applications and the growing usefulness of this technology.

## 8. Regulation Concerning 3D Printing for Pharmaceutical Manufacturing

There are numerous research articles and patent applications pertaining to the medical or pharmaceutical applications of 3D printing. However, to date, no regulations have provided recommendations or guidelines for the use of 3D printing in pharmaceutical production. In 2017, the USFDA issued “Technical Considerations for Additive Manufactured Medical Devices” guidance [116]. This guideline describes the FDA’s recommendations for 3D-printed medical devices from device development to process validation and acceptance activities. This guidance discusses numerous factors to consider while designing and manufacturing a medical device, as well as testing and labeling regulations. The guidance also recommends validating associated procedures to provide a high level of assurance, as has traditionally been the case. In addition, device validation documentation must correspond to the quality system regulation standards applicable to the device. The validation of the process is necessary to ensure and maintain the quality of all devices and their components produced in a single build cycle, between build cycles, and between machines when the outputs of a process (i.e., output specifications) cannot be changed by subsequent inspection and testing. In addition, software must be certified according to an established standard for its intended application. This guideline can be adapted to be used in 3D printing at hospitals and medical centers or at the point of care.

However, as of 2022, the FDA has not issued any guidance or regulations regarding the use of 3D printing in drug manufacturing. A discussion paper was published in December 2021, and the agency announced its intention to issue drafts and final guidance documents based on the feedback it receives. Organizations have requested that the FDA develop rules or guidelines describing how it defines device risk and regulates products based on this definition [117].

The European Union’s European Medicines Agency (EMA) also plays a significant role in the development of guidelines and standards governing this emerging field [118]. Similarly to the USFDA, the EMA has not yet issued any regulations regarding 3D-printed dosage forms. However, the manufacturers of 3D printers used in the production of medical devices and drugs must conduct a risk assessment to ascertain the health and safety requirements applicable to the machinery (Machinery Directive 2006/42/EC) [119]. Nevertheless, the EU legal framework is technologically neutral and does not mandate specific technical solutions for the design of products. Therefore, manufacturers may utilize a variety of technical solutions to satisfy these essential requirements.

## 9. The Challenges around the Universal Application of 3D Printing in Hospitals and Pharmacies

There are currently several barriers that make it difficult for hospitals and pharmacies to deploy broad 3D printing, despite the fact that the technology has a great deal of potential in the medical field. These hurdles include the following factors.

### 9.1. Regulatory and Safety Concerns

Three-dimensional printing in healthcare involves the production of medical devices, implants, and medications, which raises regulatory and safety concerns. The challenge of ensuring the safety and effectiveness of these products is significant. To ensure that 3D-printed healthcare products satisfy the necessary quality, safety, and performance requirements, regulatory bodies must establish clear guidelines and standards [120].

### 9.2. Quality Control and Standardization

Standardization and quality control are indispensable to the healthcare industry. Variability is introduced into the manufacturing process by 3D printing, which can affect the quality and efficacy of printed objects. It is essential to standardize the processes, materials, and quality control measures of 3D printing in healthcare to ensure reliable and reproducible results [121].

### 9.3. Material Selection and Biocompatibility

Material selection is crucial for 3D printing in the healthcare industry. For medical devices and implants to be biocompatible, they must not cause adverse reactions or damage to the patients. Identifying and validating materials that satisfy the biocompatibility requirements for various medical applications continues to be difficult [122].

### 9.4. Intellectual Property and Copyright Issues

Three-dimensional printing permits the duplication of objects, which raises concerns about the infringement of intellectual property rights and copyrights. The ubiquitous availability of 3D printing technology makes it more difficult to safeguard the intellectual property of medical devices and pharmaceuticals. Developing appropriate legal frameworks to resolve these concerns is necessary to ensure that the technology is used fairly and ethically [123].

### 9.5. Cost and Scalability

Although 3D printing has the potential to reduce healthcare costs by facilitating on-demand production and customization, the initial investment in 3D printing equipment and materials can be costly. In addition, it can be difficult to scale 3D printing to satisfy the needs of large healthcare facilities or the general population. For widespread adoption, overcoming these cost and scalability barriers is essential [122].

### 9.6. Training and Expertise

The operation of 3D printers and the design of printable objects require training and specialized knowledge. It is essential to train healthcare professionals, including clinicians, pharmacists, and technicians, to utilize and benefit from 3D printing technology. Incorporating 3D printing education into healthcare curricula and providing opportunities for ongoing training are required to develop the required expertise [62]. 

Despite these obstacles, ongoing research, technological advances, and collaborations among industry, regulatory bodies, and healthcare institutions are progressively overcoming them. In the future, the universal implementation of 3D printing in hospitals and pharmacies may become more feasible, providing patients with personalized medicine, improved treatment outcomes, and enhanced healthcare delivery.

## 10. Conclusions and Future of 3D Printing in Pharmaceutical Applications

Three-dimensional printing has the potential to revolutionize the pharmaceutical industry by enabling the production of customized and personalized products that are tailored to the specific needs of individual patients. Three-dimensional printing can potentially be used to produce a wide range of drug delivery systems, such as tablets, capsules, patches, and implants, in hospitals and pharmacies to enable the administration of medication in a controlled and targeted manner. This can improve the effectiveness and safety of medications and reduce the risk of side effects. Moreover, 3D printing can potentially reduce the need for large-scale production and inventory management and enable the production of small batches of products on demand, which can improve the efficiency and flexibility of hospitals and pharmacies.

In the near future, 3D printing is likely to be introduced in hospitals, pharmacies, or even houses for drug dispensing. Therefore, pharmacists, who are the main users, have to prepare their knowledge and skills for this future area of work [124]. Most importantly, the ability to implement these scenarios can be realized by addressing in-depth practical challenges that range from safety-first (from the patient’s perspective) to everyday practice (from the perspective of healthcare workers). Lastly, guidance concerning how to prepare or manufacture medicine via 3D printing remains unclear. Therefore, significant changes must occur considering the current regulations and perspectives of all related professionals or public parties [125]. 

## Figures and Tables

**Table 1:** Advantages and limitations of 3D printing in pharmaceutical applications.

Advantages	Limitations
Customization and personalization—3D printing allows for the production of customized and personalized products that are tailored to the specific needs of individual patients to improve the effectiveness and safety of the treatment [59,60,61].	Regulation and quality control—Regulatory and quality control issues must be addressed to ensure the safety and efficacy of 3D-printed pharmaceutical products [59,62].
Complex structures and geometries—3D printing allows for the production of complex structures and geometries that are not possible with traditional manufacturing methods and that can enable the development of new and innovative drug delivery systems [1,13,14,26,63].	Material selection and compatibility—The selection of materials suitable for use in 3D printing in the pharmaceutical industry is limited, and compatibility issues exist with certain drugs or formulations [59].
Cost and efficiency—3D printing can potentially reduce the cost and increase the efficiency of the manufacturing process by enabling the production of small batches of products on demand and reducing the need for large-scale production and inventory management [63,64].	Scaling up production—Technical challenges exist in scaling up the production of 3D-printed products to meet the demand of the market [59,65].

**Table 2:** Summarized reports or research of practical application of 3D printing for pharmaceuticals in hospitals and pharmacies.

Application	3D Printing Technique	Implementation Place	Reference
Personalized isoleucine chewable tablet for maple syrup urine patient (rare metabolic disorder)	Semi-solid extrusion	Clinic University Hospital, Spain	[67]
Polypills for polypharmacy patients	Fused deposition modelling	Zealand, Denmark	[68]
Investigate the influence of the shape, size, and color of different placebo 3D-printed tablets	Fused deposition modelling	University College London, UK	[69]
3D printing for personalized medicines	NA	Industry, community pharmacies, hospital pharmacies, compounding facilities, and in patients’ homes	[70]
Oral personalized 3D-printed medicines for pediatric patients	NA	Hospital and community pharmacies, Finland	[59]
Prepare paediatric medicines with enhanced palatability	Fused deposition modelling	NA	[71]
Prepare accurate, safe, and convenient subdivided drug (spironolactone). The 3D-printed tablets of 2 mg have been used in clinical inpatients and were accepted by pharmacists, nurses, and patients.	Semi-solid extrusion	Grade III-A hospital in China	[72]
Prepare polypill capsules with bespoke release patterns for multiple drugs to solve polypharmacy problems.	Fused deposition modelling	NA	[73]
Determine and compare the relative bioavailability of a novel oral 3DP rapidly disintegrating or fast-melt tablet containing levetiracetam in healthy male and female subjects.	Binder jetting	Algorithme Pharma Inc., Montreal, Quebec, Canada	[74]
Create orally disintegrating printlets suited for patients with visual impairment	Selective laser sintering	NA	[75]
Manufacture intraoral films and incorporate Braille characters in the available area for visually impaired patients	Fused deposition modelling	NA	[76]
Manufacture a tailored oral drug delivery device with a customizable design and tunable release rates in the form of a mouthguard and, subsequently, evaluate the performance of this system in the native setting in humans	Fused deposition modelling	NA	[77]
Prepare clonidine hydrochloride-loaded wearable personalized 3D printed orthodontic retainers for local sustained-release of drugs	Fused deposition modelling	NA	[78]
Investigate the preference of children for 3D printed tablets (Printlets™) prepare from four different 3D printing techniques	Digital light processing, selective laser sintering, semi-solid extrusion, and fused deposition modeling	NA	[79]

NA: data not available.

## Data Availability

Not applicable.
